# Supplementary material for: Are carbon emissions trading and green financial instruments synergistic? -Comprehensive quantitative research based on content analysis
Source: PLoS One. 2024 Mar 7;19(3):e0298601. doi: 10.1371/journal.pone.0298601 (PMC10919601; doi:10.1371/journal.pone.0298601)
Supplement: S1 Data — (ZIP) [file pone.0298601.s001.zip › Data/地级市Z_F工作报告.zip!/┴╔─■/╜⌡╓▌╩╨/└┤╫╘╣1⁄2╓┌║┼í╛┬φ┐╦╩2╛▌═°í┐_2007.docx]

各位代表：

　　现在，我代表市政府向大会报告工作，请予审议，并请政协委员提出意见。

　　一、2006年工作回顾

　　2006年是我市实施“十一五”规划的第一年，也是实现“三年大变样”战略目标的关键一年。在市委的正确领导下，全市上下坚持以邓小平理论和“三个代表”重要思想为指导，全面落实科学发展观，按照市委九届十次全会和市第十次党代会精神，以锦州湾整体开发为突破口，进一步深化改革扩大开放，加快实施工业倍增、农业升级、城市南扩、建设商贸中心、打造文化名城、构建和谐锦州六大举措，辽西沿海经济区中心城市建设取得突破性进展，较好地完成了市十三届人大四次会议确定的各项任务。

　　初步测算，全市地区生产总值实现460亿元，按可比价格计算，增长16%。三次产业比重调整到21：39：40。全社会固定资产投资完成121.7亿元，增长30.4%。地方财政一般预算收入完成20.6亿元，增长19.4%。实际利用外资9558万美元，增长43.8%；实际利用域外资金45亿元，增长40%。出口创汇完成6.3亿美元，增长16.8%。城镇居民人均可支配收入9981元，增长12.6%；农民人均纯收入4165元，增长11.7%。居民消费价格总水平增幅1%。城镇登记失业率控制在5.3%以内。

　　一年来，我们主要抓了以下十个方面的工作：

　　（一）加速沿海经济带建设，锦州湾整体开发实现新突破

　　深入贯彻省委、省政府“五点一线”开放战略，“一港、一区、一带、一面”建设取得实质性进展。

　　港口建设成果明显。25万吨油泊位竣工试运营，205、206杂货泊位及主航道浚深等工程进展顺利，203泊位疏浚和207、208集装箱专用泊位建设完成。吞吐量完成3150万吨；集装箱运输36万标箱，增长79.1%。完成了锦州港总体规划修编工作，西部港区开发前期准备工作有序推进。锦州港再度入选“新财富100家最有成长性上市公司”。

　　开发区建设快速推进。西海工业区被列为省“五点一线”重点发展区域之一。西海重化工业园和白马综合工业园基本实现了“七通一平”。东方集装箱、兵吉燕低碳脂肪胺、华能不锈钢焊管、圣合科技电子等12个项目竣工投产，玥宝塑业建筑墙板、佳岚汽车零部件等21个项目开工建设，国家石油战略储备基地、北方煤化工、中电投锦赤铁路、中海油炼化一体化等一批项目前期工作顺利推进。白沙湾行政生活区规划编制工作如期完成，区内道路网建设已经启动。锦州港海滨家园等10个房地产项目相继开工，建筑面积达到51.5万平方米。全区固定资产投资增长30%。

　　凌海百里工业带建设全面启动。以56公里滨海公路为骨架的百里工业带整体规划已经完成，娘娘宫临港产业园区（凌海经济开发区）挂牌成立。华龙焙烧工艺改造、德泰耐火材料、辽西粮食仓储基地、达莲真空冻干海产品、百通小菜等一批项目开工建设，美国UPC公司风电项目正式签约。

　　“港区带”牵动辐射作用日益凸显。锦州港、开发区、凌海沿海工业园区和工业项目延伸发展，实现了沿海经济带和黑山、北镇、义县等县（市）的统筹联动发展。

　　（二）超额完成倍增必保指标，工业经济发展再上新台阶

　　工业经济发展势头强劲，规模以上工业总产值实现642亿元，增长30.2%，提前一年完成倍增必保指标；增加值实现130亿元，增长25%。工业固定资产投资完成52.4亿元，增长46.3%。

　　重点项目全面推进。全市共有240个规模以上项目开工建设，其中1000万元以上项目162个，亿元以上项目13个。75个投资3000万元以上重点推进项目中，中石油8万吨苯乙烯、日电5万吨锰硅合金等30个项目竣工投产，华光金属化陶瓷壳、进永汽车配件等38个项目开工建设。全市亿元以上企业达到87户，5亿元以上达到13户，10亿元以上达到7户。

　　产业格局不断优化。石油化工、新型材料、农产品深加工、汽车零部件、电子信息五大重点产业实现产值564亿元，增长31.1%；机械制造、轻工纺织、医药、能源电力4个传统产业新上1000万元以上项目60个，新增产值42亿元。重新调整发展规划，确定了打造冶金和装备制造业基地的目标。汤河子工业区纳入西海工业区，享受省“五点一线”开放战略优惠政策。全力打造百亿光伏产业基地，开发区光伏产业园区一期工程正在抓紧进行，凌海多晶硅、凌南单晶硅、汤河子四氯化硅生产基地建设快速推进，“一园三基地”格局初步形成。

　　民营经济进一步壮大。西洋特肥、福山木业、金华冶炼、阳光能源、天合化工等一批民营企业迅速向规模化、集约化、现代化方向发展。规模以上民营企业达到751户，工业增加值增长50.8%，民营经济占地区生产总值比重达到69%。

　　国企改革取得重大进展。金纸改制已经完成，重型改制进入实质性操作阶段，中信集团收购铁合金国有股权工作顺利完成，华光与东芝白云真空开关管项目正式签约，矿山机械厂、焊条厂、印染厂等一批企业通过改制焕发生机。107户企业完成改制，占省考核指标的87%。

　　（三）大力推进农业升级，新农村建设呈现新气象

　　按照“生产发展、生活宽裕、乡风文明、村容整洁、管理民主”的总体要求，加快推进农村基础设施建设和各项事业发展，100个试点示范村建设取得预期成效，新农村建设实现良好开局。农业和农村经济在遭受旱灾的情况下仍然实现较快发展，农林牧渔业总产值实现180亿元，增长7%。

　　农业升级步伐加快。在结构升级上，新增棚菜面积5.9万亩，完善农业科技示范园区101个。在增长方式升级上，新建高标准养殖小区170个，新增有机绿色食品认证49个、无公害农产品认证18个。粮食仓储、供给、加工能力显著增强，继续保持省内领先地位。在经营模式升级上，不断完善农业产业化10大经营体系，规模以上龙头企业达到140户，带动农户30万户，订单农业面积达到200万亩。转移农村劳动力7万人。对56个村实施整村推进式扶贫，2.5万人实现稳定脱贫。

　　县域工业快速发展。全市各类工业园区和工贸小区达到74个，完成规模以上新扩改工业项目151个。黑山黑大、北镇沟帮子、凌海双羊和娘娘宫、义县七里河等一批工业园区不断发展壮大，国能黑山生物发电厂、五峰米糠油、光和密封垫、天宜不锈钢板等一批项目入驻园区。沟帮子经济技术开发区晋升为省级开发区，七里河工业园区进入省重点支持园区行列。

　　农村生产生活环境明显改善。完成了小凌河汇流口段河道堤防工程、绕阳河收尾工程及龙湾水库除险加固主体工程。新增灌溉面积13.2万亩，水土流失治理16.3万亩。人工造林36.2万亩，建设万树村庄115个，森林覆盖率达到23.3%。实施人工降雨（雪）作业，增加水资源2.7亿立方米。农业综合机械化水平达到58%。新建动物卫生区域监督所49个，形成了覆盖市县乡村的四级动物卫生防疫体系，实现了重大动物疫病“零控制”目标。加强基本农田保护工作，确保了重大项目建设用地。锦州中心渔港和南凌一级渔港投入使用。加大水源地保护力度，实施了大小凌河扇地防海水入侵二期工程。新建农村饮水工程74处，解决了10.4万人安全饮水问题。建设农村户用沼气池5015个。村通油路429公里，通油路村达到72.4%。公路大中修72.3公里，20个县乡客运站完成改造工程。

　　（四）加强城市建设管理，城市面貌发生新变化

　　城市南扩十大工程基本完成。渤海大道建成通车，市府广场竣工投入使用，第三医院挂牌成立，南山核心区造林任务全部完成。女儿河带状公园开工建设，绿地土方整理等基础设施工程全部完工。新建学苑路、科技路等街路8条，凌南道路网基本形成。“宝地城”、“锦绣天第”等7个房地产项目相继开工建设，完工面积突破100万平方米。滨海公路开发区段6.4公里竣工通车。

　　城市综合管理水平不断提高。深入开展全国卫生城和环保模范城创建工作。积极推进环卫体制改革，实施城市管理监督员制度，强化网格化管理和“门前三包”责任制，街路卫生状况明显改观。开展马路市场专项整治工作，规范广告牌匾，清理乱贴乱画，根治扰民烧烤，提高亮化水平，城市形象进一步好转。非物业住宅区整治任务基本完成。加大环保执法力度，关停违法排污企业32户，城市88%以上天数空气质量优于国家Ⅱ级标准。

　　老城区改造和重点工程建设顺利推进。完成了城市总体规划调整报批前期工作，制定了主城区住房、沿海城镇带等建设规划。全面完成了省政府下达的棚户区改造任务，建设回迁安置楼房33万平方米。在全省率先实施了5万平方米以下的关单棚户区改造工程，并实现了当年拆迁、当年建设、当年回迁。大修改造主要道路30条、小街小巷29条，新增城市绿地面积85.8万平方米。完成了48条街路路灯安装改造任务，主城区基本消灭了“摸黑路”。新增供暖面积130万平方米、燃气用户4265户，渤海大道公交线路顺利开通。实施了小扒沟治理改造工程，客运东站改造工程开始启动，汉口街公铁立交桥工程的拆迁、招标等前期准备工作全部就绪，大唐热电项目前期工作进展顺利。

　　（五）三产辐射功能不断增强，商贸中心建设取得新进展

　　社会消费品零售总额实现157.9亿元，增长14.5%。物流业蓬勃发展。恒大、华联、渤海物流运营良好，二期工程均已启动。恒大保税物流中心通过省政府批准，锦州港粮食现代物流项目列入省粮食物流第一批重点示范项目。现代商业设施日趋完备。引进商品流通项目65个，新建、改扩建商业项目29个，新林西路综合市场等投入运营，新华广场即将竣工开业，锦州国际会展中心项目开工建设。各类专卖店连锁配送项目发展迅速，商业网点数量达到7万个。“万村千乡市场工程”快速推进，新建、改造农家店587家，北镇窟窿台和凌海唐家蔬菜批发市场纳入全国双百市场改扩建项目，农村消费环境明显改善。深入实施质量与名牌战略，国家地域保护产品及省名牌产品达到27个，万德减震器、新日硅单晶硅被评为国家免检产品，实现了我市国家免检产品零的突破。锦州百货大楼被评为辽西地区唯一一家全国“无假货示范店”。加强和完善食品安全分工责任制，建立了“工商监管、企业自律、社会监督”的三位一体监管模式。食品、药品和生产资料市场整治成效显著。旅游业开发改造、宣传促销力度不断加大，辽沈战役纪念馆“红色旅游”品牌在全国叫响，旅游总收入增长28.1%。居民消费水平不断提升，住房、汽车等大宗商品及休闲度假、保健、文体等服务类消费持续增长。

　　（六）加大招商引资力度，对外开放取得新成果

　　坚持开放牵动战略不动摇，重视开放、支持开放、参与开放的氛围日益浓厚。韩国、香港、上海、昆山、宁波等投资说明会成果显著，意大利、日本等国家和深圳、满洲里等城市小团组经贸洽谈活动取得圆满成功。日本东芝等一批项目落户锦州，荷兰孚宝等一批在谈项目进展顺利。鼓励支持现有企业增资扩股、扩能挖潜，汉拿电机、锦泰金属、锦恒气囊等企业增资额达到全市利用外资总额的60%以上。进一步加大奖励、扶持和服务力度，帮助企业适应市场调整出口产品结构，出口创汇平稳增长。推行全程办事代理制、“绿色通道”审批制、企业登记注册“并联审批”制，行政审批公开透明程度不断提高。县（市）区公共服务中心进一步完善和规范。口岸“大通关”工作进展顺利，电子口岸及查验联合办公建设全面推开。投资环境不断优化，我市被国家统计局评为“全国投资环境百佳城市”。

　　（七）财税金融运行平稳，财政综合实力实现新提升

　　面对成品油购销价格倒挂、取消成品油出口、钼铁和钢材价格下降等诸多政策性减收因素，通过政策性退税、转让国有资产、财政贴息等方式，大力扶持重点税源企业发展，努力培植壮大税源。加大税费征管力度，全力清缴陈欠税款，堵塞征管漏洞，严厉打击偷税、抗税、骗税等行为，对乱拉税及违反政策“先征后返”进行有效遏制，基本做到了应收尽收，税费征管秩序进一步规范。强化非税收入管理，实行收缴联网管理，有效增加了政府统筹财力。进一步完善国库集中收付和政府采购制度改革，启动收支分类改革，公共财政体系逐步完善。积极争取上级资金，充分利用世行贷款和日元回收再贷款，保证了一批重点建设项目的顺利推进。优化支出结构，压缩一般性支出，调整支出预算，确保了国家收入分配改革政策的落实，保证了企业改制、社保支出、政权运转等重点支出需求。

　　金融部门对地方经济的服务作用日益增强，优势企业和重点项目得到有效支持。出台了《锦州市推进中小企业融资工作意见》，中小企业担保公司筹建工作进展顺利。切实改善金融生态环境，有效防范和化解金融风险，确保了银行信贷资金良性运转。积极争取国家开发银行政策性贷款，并已到位3.2亿元。农村信用联社改革工作有序推进，累计清收处置不良资产9.3亿元。市商业银行荣获“全国最具竞争力城市商业银行”殊荣。全市银行业金融机构本外币各项存贷款余额分别达到574.7亿元和330亿元，增长12.3%和17.3%。

　　（八）推进文化名城建设，社会事业取得新成就

　　科技、人才工作不断创新。争取国家和省级科技计划项目60项，经费实际到位1850万元。全市高新技术产业增加值增长34%，科技进步贡献率达到37%。出台了《关于提高科技创新能力加速锦州振兴的若干意见》以及《锦州市农村实用人才职称评定管理暂行办法》。成功举办了第十届中国（锦州）北方农业新品种、新技术展销会。事业单位改革试点工作顺利推进，北山宾馆等3家单位划出事业单位序列，64个乡镇完成了事业单位改革任务。教育工作扎实推进。凌河区“双高普九”、黑山县“巩固普九”通过省评估验收。建设农村九年一贯制（寄宿制）学校10所，撤并学校和教学点36所（个），完成学校危房改造4.7万平方米。学校安全工作进一步加强。锦州医学院正式更名为辽宁医学院。成功举办了古塔庙会、笔架山海会、国际民间文化节、古玩文化节、京剧票友节等大型群众文化活动。京剧《酒魂》荣获第三届全国少数民族文艺会演最高奖项――会演大奖。医巫闾山满族剪纸、黑山二人转、辽西高跷、辽西木偶4个项目入选首批国家级非物质文化遗产名录。市图书馆、少儿图书馆投入使用。广播电视节目质量大幅提高，技术设备全面升级，大型宣传活动成效显著。圆满承办了省“十运会”，开幕式“天辽地宁”受到社会各界好评，竞技比赛取得奖牌第四、金牌第五、团体总分第六的好成绩。农村防保体系建设取得突破性进展，全市89个乡镇防保机构房屋、设备基本达标,人员、经费基本到位。新型农村合作医疗工作全面启动，参合农民达到152万人，参合率达到78.2%。人口出生率和自然增长率分别控制在6.36‰和2‰以内。第二次全国农业普查工作进展顺利。外事、档案、地震等工作取得新的成绩。

　　（九）全力构建和谐锦州，群众生活水平得到新提高

　　积极拓宽就业渠道，全市城镇实名制就业安置9.6万人，完成省指标的161.1%。大力开展就业援助，594户零就业家庭有就业愿望的666人实现妥善安置。全市所有街道、社区均建立了固定劳动保障服务站（所），公共就业服务基础设施建设全部达到省政府规定标准。社会保障体系进一步健全，养老、失业、医疗、工伤、生育保险参保人数分别达到32.2万人、39.1万人、46.7万人、21.4万人、8.5万人。五种社会保险基金连年增收并实现结余，按时足额发放率和社会化发放率均达到100%。归集住房资金26.9亿元，为完善住房保障体系提供了充足的资金储备。

　　关注弱势群体，积极开展送温暖献爱心社会捐助活动，解决了8.4万户城乡困难群众特别是禽流感疫区群众的生活困难。针对“看病难、看病贵”问题，建立健全了惠民、济困医疗服务体系。完成了11所敬老院修建任务。成功举办了“慈爱锦州”大型募捐活动，为棚户区改造募集资金1134万元。城区和县（市）困难居民最低生活保障标准分别由180元和140元提高到190元和150元。全市城市和农村低保对象保障面分别达到6.4%和2.2%。社区“一站四室”建设成绩显著，服务功能进一步完善。双拥工作不断加强，军政军民关系更加密切。

　　深入贯彻实施《信访条例》，继续坚持领导包案下访制度和信访接待日制度，一批积案难案和群众关心的热点难点问题得到有效解决。积极争创“全省综治工作先进市”，平安建设工作卓有成效。保持“严打”高压态势，城乡群众安全感进一步增强。市应急联动中心正式运行，数字化三级视频监控系统相继完成。防控体系建设进入国内先进行列，警务科技处于领先地位。“四五”普法效果显著，“五五”普法全面启动。制定了《锦州市地方煤矿特大安全生产事故应急预案》等一系列专项应急预案，应急预案体系进一步完善。组织开展了5次全市安全生产大检查，安全生产形势总体平稳。

　　（十）加强政府机关建设，干部队伍展现新形象

　　坚持依法行政，制定下发了《锦州市全面推行依法行政五年规划》。严格执行人大及其常委会通过的各项决定和决议，自觉接受人大法律监督、工作监督和人民政协的民主监督，认真办理人大议案、代表建议和政协提案。全年共办理建议提案515件、人大议案2件，办复率、满意率分别达到100%和98%。强化政府机关公共服务行为管理，加大网上政务公开、投诉咨询工作力度，定期召开新闻发布会，保证了群众的知情权、参与权和监督权。严格执行《严肃机关工作纪律四项规定》，坚持对酒店和娱乐场所进行明查暗访，共处理违纪案件11起46人。“求真务实、雷厉风行、令行禁止、严细成风”十六字要求影响广泛，“锦州港精神”、“实验学校速度”、“南山绿化干劲”深入人心，涌现出一批想事、干事、成事的机关建设典型，正气进一步上升，发展合力不断增强。

　　各位代表：2006年各项工作取得的成绩，是省委、省政府亲切关怀的结果；是市委正确领导和人大、政协有效监督，各民主党派、工商联及各人民团体大力支持的结果；也是全市人民解放思想、励精图治、团结奋斗、共同努力的结果。在此，我代表市政府向全市广大干部群众，向人民解放军驻锦部队指战员、武警官兵和公安干警，向中省直各部门、各单位，向所有关心、支持锦州发展的海内外朋友以及社会各界人士表示诚挚的感谢！

　　回顾一年来的工作，我们也清醒地认识到，全市经济社会发展还存在一些困难和问题。主要表现在：县域经济发展还不平衡，有的县（市）甚至滞后于全省平均水平，农村整体经济实力亟待快速增强；地方工业在工业总量中的份额还不突出，对全市经济贡献率偏低，产业结构调整和增长方式转变的任务依然艰巨；财政保障能力有限，由于工资支出、社保支出等刚性支出的增加，不得不靠调整预算等措施来维持正常运转；政府机关作风建设仍需不断加强，职能转变任务仍然较重，依法行政能力还需要进一步提高。这些困难和问题，都需要我们在今后的工作中切实加以解决。

　　二、2007年工作安排

　　各位代表：今年是实现锦州“三年大变样”奋斗目标的决战之年，也是加速辽西沿海经济区中心城市建设的关键一年。我市发展有动力也有压力，有机遇更有挑战。全市上下一定要以邓小平理论和“三个代表”重要思想为指导，深入贯彻省第十次党代会、省委十届二次全会暨全省经济工作会议精神，以科学发展观统领经济社会发展全局，按照市第十次党代会和市委十届二次全会暨全市经济工作会议的部署，坚持既定的工作目标和思路不动摇，大力推进改革开放和科技创新，加快结构调整和增长方式转变，加强资源节约和环境保护，促进社会事业全面繁荣，着力解决民生问题，确保“三年大变样”奋斗目标顺利实现，努力为辽西沿海经济区中心城市建设奠定更加坚实的基础。

　　2007年经济社会发展的主要目标：地区生产总值增长15%，其中一二三产业增加值分别增长8%、18%、16%；万元地区生产总值能耗降低5%以上；主要污染物排放总量减少4%以上；全社会固定资产投资增长23%；地方财政一般预算收入增长15%；实际利用外资增长20%，域外招商引资增长33%；出口创汇增长10%；社会消费品零售总额增长12%；物价总水平涨幅控制在3%以内；城市居民人均可支配收入增长10%，农民人均纯收入增长9%；城镇登记失业率控制在5.6%以内。

　　围绕上述目标和要求，重点抓好以下十项工作：

　　（一）全力推进锦州湾整体开发，构筑沿海发展新格局

　　强化“五点一线”沿海开放机遇意识，加大“一港、一区、一带、一面”建设力度，努力在基础设施和项目建设上实现突破性进展。

　　加快建设广辟市场，增强锦州港实力。继续加大投入，积极推进进港主航道浚深及配套设施建设，创造条件建设第三、四港池矿石泊位、煤炭泊位、油品泊位。抓紧做好锦州港总体规划修编报批工作，加强与大连港的合资合作，加快西部港区一期开发步伐。大力开拓腹地货源市场，积极挖掘本地货源潜力，确保年吞吐量增长10％以上。

　　完善功能积极推介，增强开发区引力。按照重化工产业和重化工接续产业发展方向，加快西海和白马工业园建设进程，增强项目承载能力。进一步完善规划，高标准建设白沙湾滨海行政生活区。认真做好保税物流中心和区港联动试点申报工作，努力争取开发区年内晋升为国家级开发区。有针对性地组织好招商推介活动，努力吸引更多的投资者关注开发区、落户开发区。实际利用外资力争突破5000万美元。

　　科学规划快速推进，增强百里工业带活力。加快完善规划，合理调整产业布局。积极拓宽融资渠道，加大基础设施建设力度，及早实现娘娘宫临港产业园区起步区“七通一平”。搞好项目包装和投资论证，有效吸引资金和项目，尽快树立形象、形成规模。

　　密切合作协调运转，增强“港区带”联动张力。通过基础设施建设、项目延伸等把临港工业、沿海经济与腹地发展紧密结合起来，努力构筑“港区带”与黑山、北镇、义县等县（市）以及广大腹地优势互补、良性互动的发展新格局。

　　（二）积极打造新型产业基地，提高工业经济运行质量和效益

　　实现重大项目引进建设的突破。坚持以重大项目拉动工业经济增长，全力推进重点工业项目的引进和建设。积极促进天合化工烷基苯磺酸、玥宝塑业建筑墙板等60个续建项目尽快投产达效，中石油锦州石化150万吨氢裂化、金纸25万吨文化纸等40个项目如期开工建设。抓紧协调各方，力促国家石油储备基地、大唐热电、北方煤化工、元成玉米二期等一批项目及早开工，千万吨炼厂和百万吨乙烯、阿迪达斯运动鞋等一批在谈项目取得实质性进展。力争新增规模以上企业100户，亿元以上企业20户；规模以上工业总产值、增加值和工业固定资产投资均增长20%。

　　实现新型产业基地建设的突破。继续按照“324”产业格局优化工业结构，重点发展石化、汽车零部件、光伏、特种铁合金、焊接材料、农产品深加工等10大新型产业基地。加大对光伏产业的扶持力度，加快编制光伏产业园区建设工程可行性报告，适时出台相关优惠政策，有效推进新世纪石英玻璃1000吨多晶硅、金华冶炼500吨多晶硅、华昌光伏100兆瓦太阳能电池等项目建设，力争将我市光伏产业基地纳入国家“十一五”火炬计划特色产业基地。充分利用“五点一线”优惠政策，加速整合开发汤河子工业区，努力将其打造成为冶金和装备制造业基地。

　　实现民营企业发展规模的突破。鼓励民营企业进行技术创新和新产品研发，培育打造知名品牌。引导有条件的民营企业实行股份制改造，吸引社会资本，实现企业股权多元化。重点支持沈宏、元成、华龙、汉拿等龙头企业做强做大。民营经济占地区生产总值比重达到72％。

　　实现国有企业改制的突破。充分利用国家实施政策性破产五年规划的机遇，全面完成省市企业改制考核指标。全力抓好金纸、铁合金等改制企业的后续工作，妥善处理好债权债务、股权转让、职工安置等问题。积极推进公用事业改革。

　　（三）切实增强县域经济实力，全面建设社会主义新农村

　　按照农业主导型、生态家园型、文化旅游型、工业园区型、商贸服务型、扶贫开发型六种模式，扎实推进社会主义新农村建设。

　　加快发展县域工业。全面落实各项政策，加大对县域经济发展的支持力度，增强基层财政保障能力，确保县域经济在全市经济中的比重有较大提升。继续抓好工业园区建设，通过资本积累再投入和有效融资等手段，加大基础设施投入，不断完善园区功能，加速引进具有龙头带动和支撑作用的项目。依托工业园区建设，因地制宜发展优势特色产业，加速推进县域工业向规模化、集约化方向发展。

　　完成农业升级三年目标。大力发展设施农业，建设棚室5万亩、高标准养殖小区150个。完善检验检测体系，推进无公害农产品和绿色、有机食品的生产销售，培育名优品牌。着力提高农业产业化水平，规模以上产业化龙头企业达到160户。完善科技示范园区，搭建新品种、新技术进村入户平台。加强农产品市场预测，为农民提供便捷信息服务。开展科技培训，培养一批懂科技、善经营、会管理的新型农民。保持粮食加工、供应、流通在省内领先地位，购销总量达到1500万吨。加快农村劳动力向非农产业和城镇转移，新增转移农村劳动力5万人。加大扶贫开发力度，对50个贫困村实行整村推进式扶贫，移民300户，解决2万贫困人口生产生活困难。

　　进一步改善农村生产生活环境。建设病死畜禽无害化处理场和外引动物隔离场，提高防疫能力。整修河道，加固堤防，加快实施大小凌河防洪、防海水入侵二期等水利工程建设，新增灌溉面积10万亩、改洼治涝面积10万亩、水土流失治理面积12万亩。完成人工造林30万亩，建设100个绿化新村。提高农业装备水平，综合机械化水平提高1个百分点。开发云水资源，有效增加水资源总量。开展新农村、新电力服务，努力实现同网同价。继续加大水污染治理、水资源保护以及基本农田和海洋渔业资源保护力度，进一步改善农业生态环境。加速推进新农村建设“三个一”工程，建设好100个新农村示范村。加强村庄综合整治，扩大“一池三改”覆盖面，完成143个村360公里通油路工程，促进农村人居环境改善。进一步提高村务公开水平，全面推进农村民主管理进程。

　　（四）提高城市规划建设管理水平，树立滨海锦州崭新形象

　　巩固城市南扩成果。认真编制女儿河、小凌河、百股河周边概念性规划，进一步做好渤海大道绿化工作，适时启动南山绿化二期工程，高质量完成女儿河公园三期工程，积极促进吉祥新村等一批房地产开发项目尽快开工，着手规划云飞南街小凌河桥和女儿河桥，继续完善凌南各类市场建设，开工建设25公里滨海公路路基改造和桥梁建设工程。

　　加快老城区改造和重点工程建设。改造南京路、重庆路、锦朝路、锦义路等24条街路，拓宽通往北普陀山的重观路。采取政府主导和市场化运作相结合的办法继续推进棚户区改造进程。加大城市绿化投入，新增绿地面积75万平方米。开工建设东湖森林公园一期工程、中水回用工程和汉口街公铁立交桥，做好污水处理厂二期工程规划，启动自来水二级泵站接收改造工作。完成198.8公里公路大中修工程和高天铁路增容改造项目，力争客运东站改造工程年内竣工、锦凌水库项目国家报批工作取得新的进展。

　　提高城市综合管理能力。坚持建管并举，加快环卫、城管体制改革进程，建立健全城市长效管理机制。继续完善网格化管理，不断推进“门前三包”向街道、社区和小街小巷延伸。治理城市“疮疤”，扩大拆墙透绿成果，提高美化亮化档次。加大整治力度，进一步规范牌匾、广告和店面管理，依法规范和取缔马路市场。搞好城市建筑垃圾处理和危险废物综合利用工程建设。促进城市卫生、空气、生态等环境质量尽快达标，争创全国卫生城和环保模范城。

　　（五）构建新型流通服务体系，加快区域商贸中心建设

　　发展现代服务业。以建设辽西区域性商贸中心为目标，开放、整合流通资源，大力发展以物流、信息、配送、会展、咨询、中介、理财等为重点的新型服务业。积极推进恒大物流及保税中心、渤海物流和华联物流二期等项目建设，逐步形成集高效运输网络、电子配送体系、现代仓储设施、大型购物中心、连锁综合超市为一体的先进流通服务业态。完善配套服务设施，进一步扩大车展、房展、古玩展等展会规模。

　　提升传统服务业。坚持政府引导、市场运作、社会投资相结合，加快传统服务业的改造升级。积极引进沃尔玛、家世界等国际知名企业，加快宏发建材商城三期、凯斯特电器商城等项目建设，推进餐饮、休闲、维修业态创新和结构优化，全面提升传统服务业档次和水平。加大重点旅游产品开发建设和宣传推介力度，打造品牌，增强效益，实现旅游收入增长13.5%。

　　完善农村市场体系。继续实施“万村千乡市场工程”，加强县乡中心店建设，新建改造“农家店”150家，增强商品下乡配送能力。启动“信福工程”，构建商业信息服务体系。增强北镇窟窿台蔬菜批发市场、黑山励家杂粮市场等大型市场的集散辐射功能，升级改造北镇青堆子鲜蛋批发市场等10个农产品市场。

　　（六）坚持实施对外开放战略，切实加大招商引资力度

　　强力推进招商引资。坚持引资、引智并举，有效推进优势产业、改制企业、重大基础设施以及商贸、文化、公用事业等领域的招商引资。切实搞好项目筛选和包装，确保市级包装精品项目不少于40个，各县（市）区不少于10个。以韩国、日本、香港、台湾等国家和地区以及长三角、珠三角为重点，采取小规模、多批次、专业化方式，积极开展招商引资活动。鼓励扶持有能力的企业进一步增资扩股、扩大规模。加强各县（市）区、市直部门和企业的协调配合，立足客商需求，整合招商资源，实现政策、土地、能源、信息互补共享。

　　优化结构扩大出口。坚持市场多元化、以质取胜和科技兴贸战略，在促进中石油锦州石化公司、沈宏、华宇等出口龙头企业扩大出口的同时，增加高新技术产品和农产品出口份额。支持有条件的企业扩大境外投资合作，带动产品、技术出口和劳务输出，尽快形成新的出口增长点。

　　切实改善投资环境。全力推进行政审批全程代理制度，简化审批手续、降低审批门槛、提高审批效率。坚决制止乱收费、乱罚款、乱检查、乱摊派等现象，切实维护投资者和企业的合法权益。建立外商投资企业季度协调会制度，帮助企业解决实际困难。依托传媒资源教育市民自觉支持开放、参与开放，积极营造“亲商、扶商、安商、富商”的社会氛围。

　　（七）认真做好财税金融工作，不断提升财政运行质量

　　克难点做好财政增收工作。综合运用贷款贴息、财政补助、信用担保等现行政策，支持重点项目建设，培育壮大支柱企业，建立互补型财源。加强对重点企业、重点税种的有效监控，寻求新的税收增长点。坚决杜绝乱拉税源现象，严厉打击涉税违法行为，实现依法治税、依率计征，堵塞跑冒滴漏，努力实现财政收入同地区生产总值的协调增长。规范土地出让金等非税收入管理，加强社保基金等各项基金收入的征管。推行“乡财县管”等财政管理措施，清理机关事业单位资产，整合统筹财力，确保财政管理健康有序。进一步理顺财政管理体制，切实加强市本级财源培养。

　　保重点优化财政支出结构。强化预算约束，严格预算管理。树立过紧日子思想，压缩各类一般性支出，确保国家收入分配改革政策的落实，足额安排机关事业单位职工工资、离退休费，保障农业、教育、科技、文化、卫生和生态建设等重点支出以及政权建设、社会稳定方面的支出。进一步推进收支两条线和支出绩效评价等项改革。加强审计监督，提高财政资金使用效率和效益。

　　促合作发挥金融服务作用。继续谋求与国家开发银行的深度合作，加强银政联手和银企合作，加快组建中小企业担保公司，切实加大金融对优势产业、重点项目、中小企业和民营经济发展的支持力度。加强信用社区建设，不断改善全市金融生态环境。推进农村信用社改革，加快市商业银行建立现代企业制度步伐。吸引外资银行在我市设立机构、开展业务。支持保险业规范健康发展，拓展农村保险市场。

　　（八）充分发掘科技文化潜力，促进社会事业协调发展

　　加快科技发展，提高教育水平。建立健全科技创新体制和运行机制，引导扶持重点行业和企业进行产品与技术创新，发展高科技特色农业，提高地区经济增长的科技含量。全市高新技术产业产值增长25%以上。拓展培养、吸引、使用人才领域，加强高级研发人才、复合型人才和技能型人才队伍建设。加强教育教学管理，整合教育资源，推进义务教育均衡发展，大力发展职业教育。改造学校危房3.2万平方米，建设农村九年一贯制（寄宿制）学校8所以上。加快“农远工程”建设，农村中小学全部实现“校校通”。确保古塔区通过省“双高普九”评估验收。

　　打造文化名城，发展群众体育。进一步活跃城乡文化氛围，继续办好古塔庙会、北方农展会、笔架山海会、国际民间文化节、古玩文化节、京剧票友节等大型节庆文化活动。打造文化精品，争取京剧《酒魂》进入国家舞台艺术精品工程。加强文化市场管理，积极开展以网吧为重点的专项整治活动。继续推进广播电视“村村通”工程，努力完成预期任务目标。加快群众体育基础设施建设，提高竞技体育水平。继续深化文化体制改革，不断壮大文化产业。

　　完善公共卫生体系，做好人口计生工作。强化城镇二级医疗机构服务体系建设，发展城市社区卫生服务，推进社区医疗机构达标。加强农村卫生工作，完善新型农村合作医疗制度。巩固农村防保体系建设成果，推进乡镇卫生院上划县（市）管理工作，完成乡镇卫生院国债项目建设任务。推进市传染病院与结核病院的资源整合工作。进一步落实农村部分计划生育家庭奖励扶助政策。全市人口出生率和自然增长率分别控制在8.1‰和3.19‰，切实提高人口素质。

　　（九）着力提高群众生活质量，推进和谐锦州建设进程

　　千方百计扩大就业。落实就业再就业扶持政策，完善公共就业服务体系，强化职业技能和创业培训。继续做好“零就业家庭”和大龄人员等困难群体就业援助，以及下岗失业人员、高校毕业生、城镇新增劳动力和退役军人的就业安置工作，全面解决并轨历史遗留就业问题。实现实名制就业6万人，其中安置大龄就业困难人员5000人。实施普惠制培训3万人，劳务输出3万人。

　　切实关心百姓生活。继续扩大保险覆盖面，养老、失业、医疗、工伤、生育保险人数分别达到32.5万人、39.5万人、47万人、22万人、8.5万人。建立城乡一体的最低生活保障制度。完善救助体系，整合社会资源，实行救助联动，帮助特困家庭解决就医看病、子女上学、冬季取暖、住房困难等实际问题。妥善解决老复转军人、老劳模以及残疾人的基本生活。做好拥军优属工作，努力争创全国双拥模范城。

　　确保社会和谐稳定。严格落实信访工作领导责任制，完善协调、预警和绩效考评机制，努力化解社会矛盾。加强社会治安综合治理，积极争创“全省综治工作先进市”。警力向社区和村屯下沉，严厉打击各种违法犯罪，切实增强城乡群众的安全感。充分发挥城市应急联动中心作用，妥善处置各类突发事件。继续推进“五五”普法，扩大法律服务领域，完成国债资金支持基层司法所建设任务。落实安全生产责任追究制度，加大执法力度，及时消除安全隐患。加强综合监管体系建设，开展食品、药品以及生产资料市场等专项打假活动，确保群众切身利益不受损害。落实民族宗教政策，依法加强宗教事务管理。

　　（十）大力加强政府自身建设，不断提高依法行政能力

　　强化职能转变，提高服务能力。进一步转变政府职能，切实把工作着力点转到加强经济调控、创造良好环境上来，转到促进社会事业发展、建设和谐锦州上来。抓好各级公共服务中心建设，强化公共服务和社会管理职能。推进政务公开，深化行政审批制度改革，规范审批行为，提高服务效能。

　　强化依法行政，提高执法能力。认真落实《锦州市全面推进依法行政五年规划》，深入实施《公务员法》，进一步规范执法行为。切实贯彻人大及其常委会决定、决议，完善工作报告制度，自觉接受人大依法监督、政协民主监督和人民群众社会监督，确保做好人大议案、代表建议和政协提案的办理工作。广泛听取各民主党派、人民团体的意见和建议，支持工会、共青团、妇联等群团组织开展工作。

　　强化作风建设，提高执行能力。弘扬正气，营造环境，进一步激发广大干部群众脚踏实地、负重奋进的干劲，百折不回、锲而不舍的韧劲和不达目的、誓不罢休的拼劲，以扎实的工作作风和饱满的精神状态共同推进事业的发展。实行工作目标责任制，深入开展效能监察，切实解决不作为和乱作为等问题。继续严格执行《关于严肃机关工作纪律的四项规定》，确保政府系统令行禁止、严细成风，反应快速、运转高效。

　　各位代表：回顾过去，令人自豪；展望未来，催人奋进。让我们在市委的坚强领导下，紧紧依靠全市人民，埋头苦干，奋力拼搏，确保“三年大变样”目标的顺利实现，努力为滨海新锦州建设做出新的更大的贡献！
